# Supplementary material for: Learning curve and surgical outcome of robotic assisted colorectal surgery with ERAS program
Source: Sci Rep. 2022 Nov 29;12:20566. doi: 10.1038/s41598-022-24665-w (PMC9709162; doi:10.1038/s41598-022-24665-w)
Supplement: Supplementary file 1 — Supplementary Information 1. [file 41598_2022_24665_MOESM1_ESM.zip › LSC Raw data-Ñ[▒K/117/O_ñw╝╨░O▒Kñσ.pdf]

正式摘要(電子簽章：000000210541394)

請選擇...reporter

臺 中 榮 民 總 醫 院

Operation Note

[redacted]

- 手術日期：2021/02/18
- 手術醫師：VS 蔣鋒帆，CR 林陳彥，R 林志安
- Anesthesia：GE
- Position：Lithotomy
- Aseptic agents：Betadine Aqua，Zephnan

Diagnosis

- Pre-operative diagnosis : CA OF RECTUM,L/3,POSTERIOR SP CCRT
- Post-operative diagnosis : Lower rectal cancer s/p CCRT s/p laparoscopic TME + loop ileostomy

Surgical Indication

- For removing the primary tumor

Operation

- 1. Laparoscopic TME with loop ileostomy
  2. Intraoperative colonoscopy : (-)
  3. High ligation (+) : At IMA root. IMV and left colic artery were also ligated.
  4. EndoGIA (60-4.1) X3 + EEA (33 mm) were applied
  5. 3-O V-Loc was applied for repair the rectal stump site defect
  6. Proximal cut end: > 10 cm, distal cut end: 0.2~0.5 cm
  7. Loop ileostomy was created and fixed over RLQ of abdomen (About 20 cm prior to cecum).
  8. One 12 Fr. penrose drainge tube was placed from LLQ of abdomen and inserted into the pelvic cavity
  9. The colon was not taken down from the splenic flexure site

Finding

- 1. A 2.5 x 3 cm, ulcerative tumor at lower rectum. (DRE : About 3~4cm FAV)
  2. Adhesion was noted between the S-colon and the abdominal wall
  3. No other gross tumor nor polyps was found
  4. There was no obvious visible liver metastasis, no visible peritoneal seeding.
  5. No obvious ascites was noted over the pelvic area.
  6. Bilateral doughnuts : Not intact. Defect over the rectal stump was noted s/p suture repair.
  7. Air leak test after anastmosis : No obvious air bubbles were found.

Procedure

- 1. General endotracheal anesthesia was induced and then the patient was placed in modified Trendelenburg
  lithotomy position. Preoperative antibiotics were given. A Foley catheter and a nasogastric tube were placed. The abdomen was prepared and draped in standard sterile fashion.
  2. A small vertical incision was made right next to the umbilicus and a 12 mm trocar was inserted into the peritoneal cavity by open method. Peumoperitoneum was established with CO2 inflation. Laparoscopy was inserted and then another 12 mm trocar was inserted over RLQ. Extra three 5 mm trochars were inserted over RUQ, LUQ and LLQ of abdomen. The operative findings were mentioned as above.
  3. Adhesion lysis was performed over the S-colon area for better visualization.
  4. The small bowel was inspected and retracted to the right by grasper. The sigmoid colon was then

- held by grasper and the peritoneum overlying the mesentery was then scored with electrocautery. The inferior mesentery artery was identified, ligated below the left colic artery with endoclips, and transected, so was inferior mesentery vein. Dissection was done above the Gerota's fascia and toward the peritoneal attachments.
 5. The arcade vessels to the sigmoid colon were also divided. Distal descending colon and upper rectum were mobilized.
 6. The colon was mobilized from retroperitoneum overlying the sacral promontory and into the pelvis. The incision was extended laterally and medially across the peritoneal reflection.
 7. The mesorectum was then mobilized using sharp dissection over the level of pelvic floor muscle. Then distal descending colon and rectum were mobilized as possible.
 8. DRE was performed at the same time to make sure the desired resection line is adequate. Tumor was located about 3~4cm FAV.
 9. Totally three linear staplers (EndoGIA 60-4.1) were used to divide the lower rectum.
 10. Incision was further extended from the mid-line laparoscopic trochar wound. The proximal colon was then taken out for carefully inspection, and to make sure it has an excellent blood supply. The mesenteric fat was gently cleared from the terminal 2-3 mm of the bowel ends.
 11. The field was isolated with moist pad and the bowel opened and sized. It was determined that a 33 mm circular stapler could be accommodated. A pursestring suture of 2-0 prolene was placed, the anvil was inserted, and then the pursestring suture was tied.
 12. The anal canal was irrigated with distil water and then the circular stapler was introduced through the anus and pierced through the mid-portion of the stapler line.
 13. The defect was noted over the rectal stump. 3-O V-Loc was applied for repairing.
 14. The proximal bowel with anvil in place was engaged with the circular stapler and the stapler was then fired. Two donuts were obtained and the distal one was submitted to pathology for margin confirmation. (Two donuts were not intact)
 15. The anastomosis was tested for leaks with irrigation solution and air introduced through the rectum. No air bubbles were noted.
 16. The possible bleeder was then checked thoroughly.
 17. The 12 Fr. penrose drainage tube was inserted from the LLQ trochar wound, which was then placed into the pelvic.
 18. The ADHESION BARRIER FILM was then placed below the central extended wound.
 19. The desired terminal ileum (20 cm prior to the cecum) was selected. A window was created under the ileum by dividing the mesentery adjacent to the bowel wall with electrical cautery carefully.
 20. The wound over the RLQ trochar site was further extended to serve as the ostomy site.
 21. The terminal ileum was then dragged out from the ostomy site carefully and a plastic bridge (Rod) was placed via the window.
 22. The fascia of mid line wound was closed with interrupted sutures of Num. 1 Vicryl.
 23. After irrigation the wound with N/S, the skin was closed with interrupted 3-0 nylon and the wound sites were covered with Medipore.
 24. The ileostomy was matured with multiple interrupted mucocutaneous suture via 4-0 Monocryl.
 25. The ileostomy was well formed and viable. An ostomy bag was placed.
 26. The patient tolerated the procedure well and was brought to the POR in stable condition
- Estimated blood loss: minimum
- 器械數量: 正確
- 紗布數量: 正確

Image

- PAINT
  - abdomen

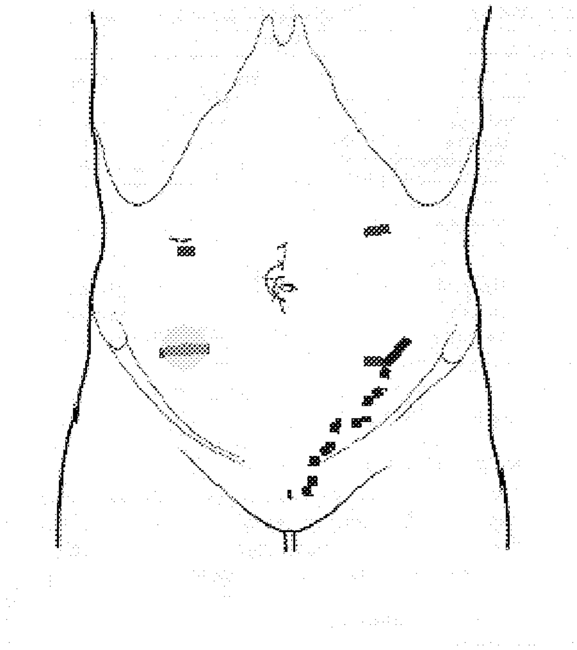

- colon & rectum

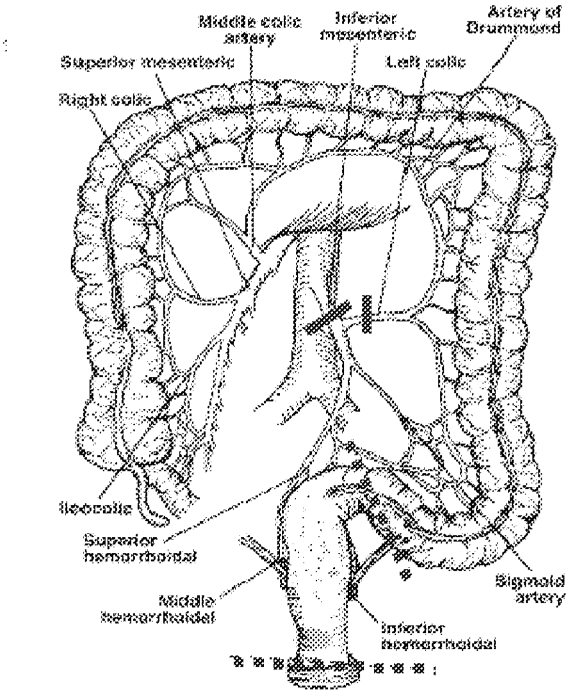

- The resected rectum

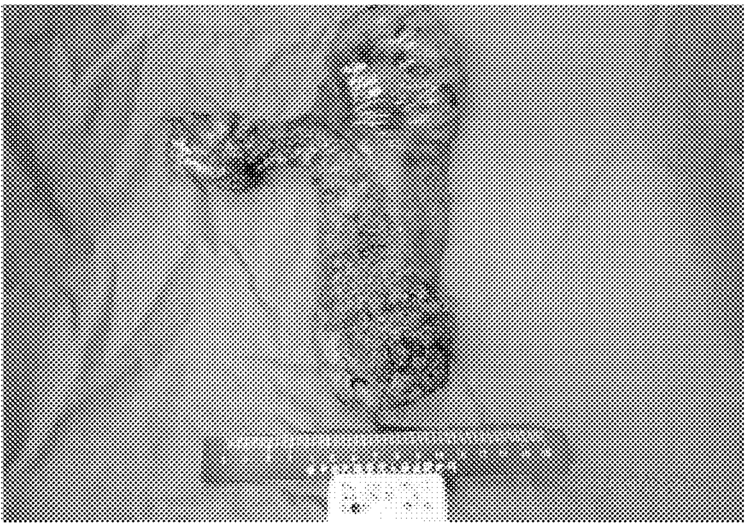

The resected rectum

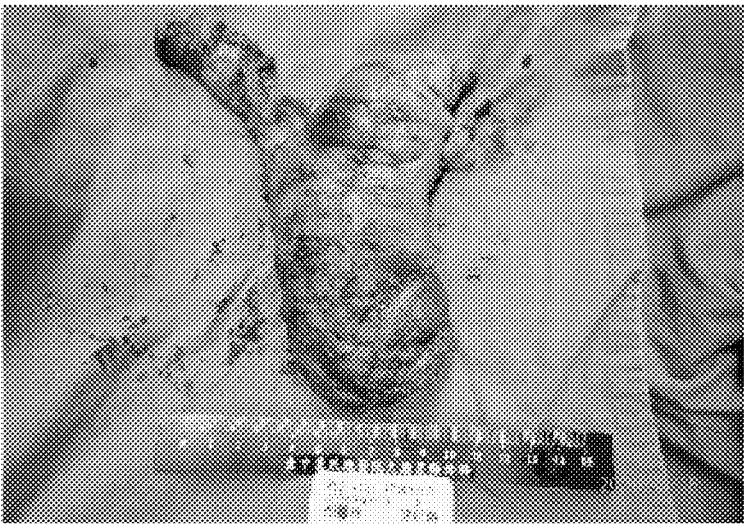

The s/p CCRT tumor could be clearly seen

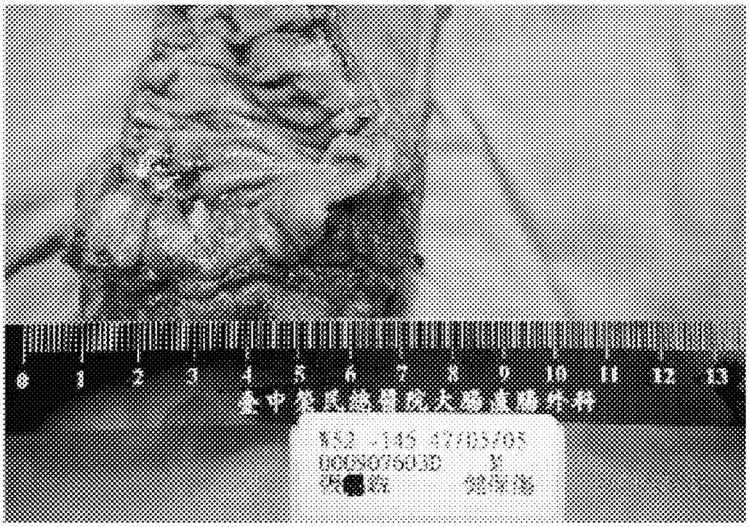

The tumor distal margin was about 0.2~0.5 cm

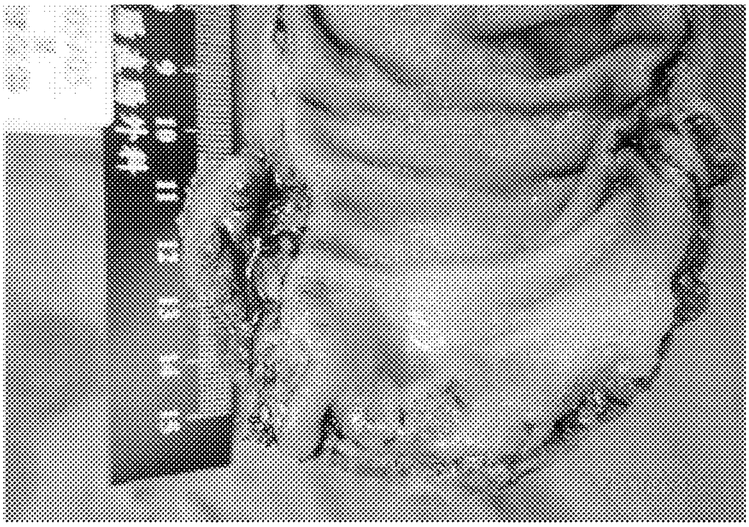

主任 2062F蔣鋒帆 / 醫師 2301H林陳彥

正式摘要**(**電子簽章：**000000210541394)**

請選擇...reporter

doctorRank:B
